# Nonlinear regulatory dynamics of bacterial restriction-modification systems modulates horizontal gene transfer susceptibility

**DOI:** 10.1093/nar/gkae1322

**Published:** 2025-01-16

**Authors:** Magdalena Djordjevic, Lidija Zivkovic, Hong-Yu Ou, Marko Djordjevic

**Affiliations:** Institute of Physics Belgrade, University of Belgrade, Pregrevica 118, Belgrade11080, Serbia; Serbian Academy of Sciences and Arts, Knez Mihailova 35, Belgrade11000, Serbia; Institute of Physics Belgrade, University of Belgrade, Pregrevica 118, Belgrade11080, Serbia; State Key Laboratory of Microbial Metabolism, Joint International Laboratory on Metabolic & Developmental Sciences, School of Life Sciences & Biotechnology, Shanghai Jiao Tong University, Shanghai200240, China; Quantitative Biology Group, University of Belgrade - Faculty of Biology, Studentski trg 16, Belgrade11000, Serbia

## Abstract

Type II restriction-modification (R–M) systems play a pivotal role in bacterial defense against invading DNA, influencing the spread of pathogenic traits. These systems often involve coordinated expression of a regulatory protein (C) with restriction (R) enzymes, employing complex feedback loops for regulation. Recent studies highlight the crucial balance between R and M enzymes in controlling horizontal gene transfer (HGT). This manuscript introduces a mathematical model reflecting R–M system dynamics, informed by biophysical evidence, to minimize reliance on arbitrary parameters. Our analysis clarifies the observed variations in M-to-R ratios, emphasizing the regulatory role of the C protein. We analytically derived a stability diagram for C-regulated R–M systems, offering a more straightforward analysis method over traditional numerical approaches. Our findings reveal conditions leading to both monostability and bistability, linking changes in the M-to-R ratio to factors like cell division timing and plasmid replication rates. These variations may link adjusting defense against phage infection, or the acquisition of new genes such as antibiotic resistance determinants, to changing physiological conditions. We also performed stochastic simulations to show that system regulation may significantly increase M-to-R ratio variability, providing an additional mechanism to generate heterogeneity in bacterial population.

## Graphical Abstract

**Graphical Abstract:**
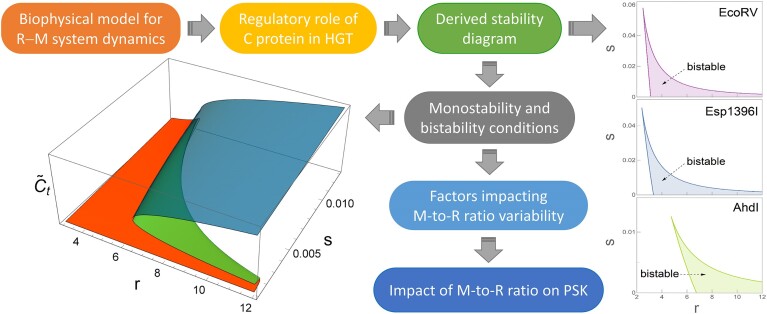

## Introduction

Restriction-modification (R–M) systems have long been recognized as a critical component of bacterial defense mechanisms (1). These systems serve as a first line of defense against invading genetic elements, such as bacteriophages, by cleaving foreign DNA while protecting the host genome through methylation (2). The dynamics of horizontal gene transfer (HGT) are profoundly influenced by R–M systems. HGT is a crucial process that allows bacteria to acquire new genes and, thereby, new functionalities, including antibiotic resistance and pathogenicity. However, the mechanisms that regulate R–M systems and, consequently, control HGT are not fully understood. The rising prevalence of strains resistant to multiple drugs, fueled by HGT of antibiotic resistance and virulence determinants, poses a growing threat to global health. Moreover, a recent study found that only a small number of Bacterial Defense Systems (BDS) (3) are associated with the reduced HGT on the scale of species evolution (4). Interestingly, Type-II R–M systems are among them, though not for all studied bacterial species (4), (5). Consequently, gaining insights into Type II R–M systems’ functioning and regulatory mechanisms is critically important from both fundamental microbiology and medical perspectives (6).

Type II R–M systems express restriction enzyme R and methyltransferase M. The same sequences that are cut by R are protected by M. This allows R–M systems to act as rudimentary bacterial immune systems since foreign DNA entering the cell is (at least initially) not methylated and is consequently cut (destroyed) by R (2). On the other hand, the host genome is methylated and, therefore, protected from cutting by R, ensuring the survival of the host genome and, consequentially, the R–M system itself. R–M systems often spread from one host bacterium to another (7). As the host genome in a na$\ddot{i}$ve bacterial cell (in which the R–M system is not yet established) is initially not methylated, it is clear that R and M expression have to be tightly coordinated during the system establishment (8,9). This coordination must ensure that the host genome is methylated (i.e., protected) before being cut by R. In a number of Type II R–M systems, this coordination is achieved by a dedicated transcription regulator called control (C) protein (10).

**Figure 1.**
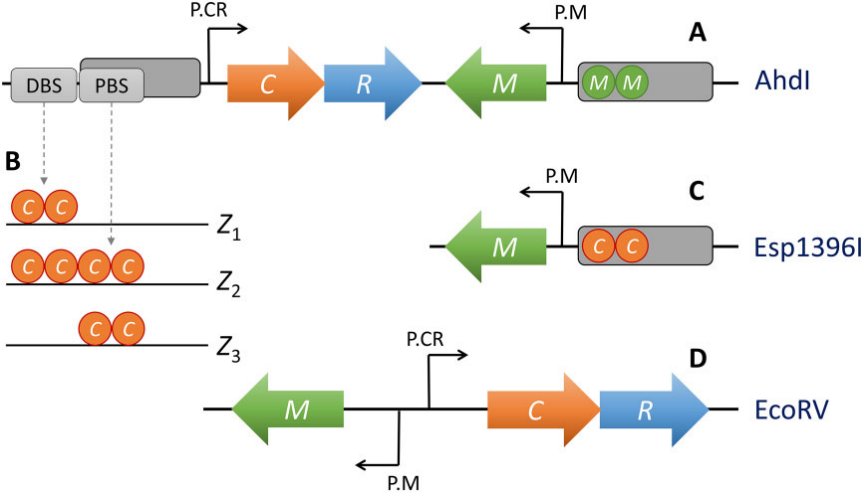
Regulation schemes for three characteristic R–M systems under C control: (**A**) AhdI (15), where a negative feedback loop through M dimer repression controls M gene promoter (P.M). (**B**) Different configurations of C protein binding to P.CR (CR promoter operon), which are the same for these systems (21), but with different interaction parameters. (**C**) For Esp1396I R–M system (22), repression of P.M is exhibited by C dimer binding. (**D**) For EcoRV R–M system (23), P.CR and P.M promoters have divergent overlapping configurations. Control of P.CR by C is the same as for AhdI and Esp1396I, i.e. as shown in B). P.M is not affected by C; however, due to overlap with P.CR, P.M is active only when P.CR is not.

Schemes of three typical and experimentally well-characterized R–M systems under the control of C protein are shown in Figure 1. For most C-controlled R–M systems, regulatory configurations for P.CR promoter are the same and are shown in Figure 1B. The C protein gene is transcribed together with that for R and regulates the P.CR promoter (i.e., its own expression and the expression of the R gene) through both positive and negative feedback loops. Positive feedback is exhibited by C dimer binding to distal binding site (DBS), which activates P.CR (configuration *Z*_1_ in Figure 1B). The binding of the C dimer to the proximal binding site (PBS) corresponds to the negative feedback, i.e. repression of P.CR (configuration *Z*_3_), which, although not observed, is included in the model for completeness. The binding of C to P.CR is cooperative, with C binding to both DBS and PBS in the form of dimer, while due to the high dimerization constant, most of the C proteins in the solution are monomers. Another cooperativity in the system is through interactions of the dimers bound at DBS and PBS (quantified by cooperativity ω). Consequently, binding of the C dimer to DBS tends to recruit the binding of another dimer to PBS so that the tetramer is formed as the repressor configuration (configuration *Z*_2_ in Figure 1B). Another property of this regulation is that the binding of C to DBS is significantly stronger (by a factor of *p*) compared to the binding to PBS. Thus, when C is synthesized, it first binds to DBS, leading to the system activation and only subsequently to PBS, resulting in promoter repression at higher C concentrations. While the regulatory architecture of P.CR for these three systems (and most other C-controlled R–M systems) is the same, biophysical interaction parameters (e.g., ω and *p* mentioned above) can be quite different, as we will discuss in more detail in the following sections.

On the other hand, P.M is regulated in different ways for different R–M systems, as can be seen from Figure 1A, C and D. Common feature of P.M regulation is that there is a strong basal transcription, which is repressed by either M itself as in AhdI (Figure 1A), C dimer as in Esp1396I (Figure 1C), or promoter overlap in EcoRV (Figure 1 D). Consequently, as R–M enters a naive bacterial cell, P.CR activity initially increases, while P.M activity decreases due to the repression of P.M through the mechanism indicated above. The initial expression of R is low due to low basal transcription activity of P.CR, i.e. low promoter leakage *s*. This provides enough time for initially strongly expressed M to protect the host genome by methylation before increasing amounts of R are synthesized by C activation of P.CR (11,12). As C increases, P.CR is repressed by C binding to PBS, which establishes a certain steady state value of R. Similarly, repression of P.M (exhibited by different mechanisms) leads to establishing a certain steady state value of M. Establishment of R–M systems in the naive bacterial host has been quantitatively reasonably well understood through a synergy of experimental and theoretical work, combining biophysical (11,12) and non-linear dynamics modeling (13,14). Specifically, our previous research has demonstrated the utility of combined biophysical and dynamical modeling in (i) interpreting transcription control measurements of R–M systems *in vitro* (15,16), (ii) elucidating the dynamics of these systems *in vivo* (17,18) and (iii) manipulating key features of the R–M system to study their impact on system dynamics (19,20).

Recent experimental studies shed light on the importance of the ratio between M and R enzymes within bacterial cells, commonly called the M-to-R ratio. Changes in this ratio are shown to significantly impact the susceptibility of bacteria to bacteriophage infections, thereby affecting the ability of R–M systems to destroy invading bacteriophage genomes (24). This shows that the HGT barrier (including the spread of pathogenic genes) can be significantly modulated by the regulation and dynamics of R–M systems, particularly by the changes in the cell’s stationary R and M levels.

While these experimental findings are significant, they also open up a number of questions regarding the regulatory processes that govern these ratios. While the establishment of the R–M system in a naive bacterial host is relatively well understood, the mechanisms driving changes in the M-to-R ratio remain unclear, even on an intuitive level (24). Specifically, it was experimentally observed that for the Esp1396I R–M system, the M-to-R ratio significantly changes (decreases) with the increase in the plasmid copy number. In the absence of regulation, a larger plasmid copy number increases the overall expression of both M and R, leading to an unchanged M-to-R ratio. As the system is regulated by C protein, the ratio may be modulated by this regulation. It is, however, intuitively hard to assess even the direction of this change with the increasing plasmid copy number, as P.CR is under both positive and negative control by C. Therefore, as C expression increases with the increase in plasmid copy number, it is unclear if this will influence R expression positively or negatively. However, M expression will be negatively affected as P.M is repressed by C. Consequently, the direction of the M-to-R ratio is unclear, and this work aims to provide a framework to systematically assess M-to-R ratio variations for different R–M systems (and external parameters), as the main system output relevant for changes in HGT barrier.

Moreover, as the system regulation involves cooperative (and consequently nonlinear) positive feedback, bistability may emerge (25), which would come as an additional layer of complexity atop changes in the M-to-R ratio. Bistability allows for two stable states of the system under the same conditions, allowing bacterial populations to diversify their responses to environmental challenges (26,27). In the case of R–M systems, bistability may lead to a subpopulation of bacterial cells with a significantly different defense activity. Actually, in the case of C-controlled R–M system PvuII, bistability in R expression was observed (28) and, as in Esp1396I, was associated with plasmid copy number. However, M expression was not considered in this study and is likely complicated due to convergent P.M and P.CR promoters that may interfere with each other, depending upon their relative spacing (29). A synthetic single-gene autoregulatory system with binding cooperativity based on phage λ repressor cI (distantly related to R–M C proteins (30)) was also found to exhibit bistability (31,32). A further complication is that bistability versus monostability likely intricately depends on the quantitative parameters of P.CR regulation since it involves both positive (promoting bistability) and negative (promoting monostability) feedback. Fortunately, a series of careful biophysical and biochemical experiments have been done for three of the C-controlled R–M systems, Esp1396I, AhdI and EcoRV (15,22,23,33–35), which is another motivation for studying C-controlled Type II R–M systems in the context of HGT barrier modulation.

In addition to the bistable switch in gene expression that arises from deterministic nonlinear dynamics, another switch-like behavior in R–M systems is induced by stochastic effects. This switch determines the ‘life or death’ fate of the cell during post-segregational killing (PSK) by Type II R–M systems (36,37). Although it is often assumed, by analogy to PSK in toxin-antitoxin systems, that PSK in R–M systems is due to the larger stability of R compared to M, this assumption has not been confirmed in cases where it has been directly tested (38,39). An alternative, more general explanation suggests that successive cell divisions lead to progressively smaller amounts of molecules, increasing fluctuations in the M-to-R ratio. This imbalance may eventually lead to autoimmunity and, consequently, cell death. While this mechanism is intuitive and plausible, it is evident that regulation of the system could significantly impact this process in two ways: First, it can influence the distribution of R and M molecules at the time of plasmid exclusion (initial distribution), which affects fluctuations during post-segregational dynamics. Second, it can modulate fluctuations in the M-to-R ratio while the system is present in the cell, potentially affecting the HGT barrier and providing protection against autoimmunity due to imbalances in the M-to-R ratio.

Given the above, we aim to develop models of P.CR and P.M regulation that are isomorphic to the biophysics/biochemistry of transcription regulation, allowing for direct parameterization from experimental data. This approach will establish a quantitative framework for analyzing various C-control systems with the following specific goals, addressed in this manuscript:

- Integrate available extensive biophysical and biochemical measurements in the model.
- Investigate the M-to-R ratio and relate it to recent Esp1396I experimental data.
- Determine whether – and under what conditions – systems can exhibit bistability.
- Analyze what system features promote or inhibit bistability.
- Stochastically model M-to-R ratio fluctuations and relate them to the dynamics of PSK.
- Assess how to guide future experiments to obtain a more extensive understanding of Type II R–M regulatory dynamics.

## Materials and methods

We develop a mathematical model describing the interactions within R–M systems, focusing on the feedback loops mediated by the control protein C. The model incorporates key biological processes such as gene transcription, protein dimerization, and DNA-protein binding, parameterized to reflect known biochemical rates and affinities. This is achieved by using a statistical mechanics (physics) model of the transcription regulation, taking into account both protein-DNA (C interactions with promoter) and protein-protein (dimerization and cooperativity in C binding) interactions (40,41). Stability analysis and bifurcation diagrams are utilized to identify the conditions under which the system exhibits bistable behavior. We aim at the minimal model, i.e. involving only experimentally observed system properties (see Introduction), but we integrate a number of experimental results for the three well-studied R–M systems (Esp1396I, AhdI, and EcoRV). The model represents deterministic (nonlinear differential equations) dynamics since system steady-state is considered, where the number of M and R molecules is high (on the order of thousands or more) (17,24) in the experiments considered here. The model also accounts for realistic system properties, such as the monomer-dimer balance in the solution, making calculations more complex but realistic. Additionally, the developed regulatory model serves as a basis for simulating system dynamics to assess fluctuations in the M-to-R ratio and their relation to PSK.

In this section, we first introduce a biophysical model of P.CR regulation, which directly applies to all three R–M systems considered here. Based on this model, we outline an analytical derivation for the system stability diagram, which applies broadly to C-controlled R–M systems with the same P.CR regulatory architecture (21). Details of these analytical derivations are provided in the Supplementary Material. Estimation of the internal parameters and, where possible, the external parameter *s* (promoter leakage) from experimental measurements is presented in the Supplementary Material. Construction of the bifurcation diagrams and the cusp catastrophe surface plot is described. Next, we provide biophysical models of P.M. regulation for all three systems. Parameter estimation for the control of both P.CR and P.M in all systems is also described. Finally, we present a computational framework for stochastic simulations of (i) the unregulated (baseline) system, (ii) the fully regulated system and (iii) post-segregational dynamics.

### Model setup

**Figure 2.**
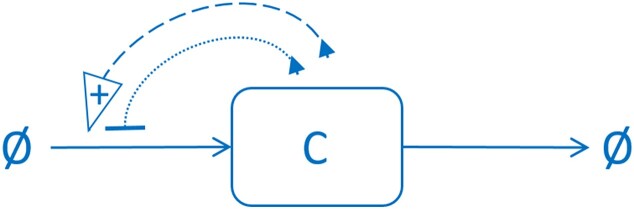
General scheme of C regulation: C protein, labeled by a rectangle, is produced (expressed) and then effectively degraded – production and degradation are labeled by two horizontal arrows, which, respectively, originate from (lead to) $\varnothing$. Autoregulation of C involves both positive (dashed arrow labeled with +) and negative (dotted line ending with −) feedback. Indicated feedback loops are a simplification of the regulatory mechanism in Figure 1B.

We start from one transcription factor (control protein “C”), which dimerizes and binds to its promoter as a dimer. This binding can lead to both positive and negative feedback, as indicated in Figure 2, in accordance with Figure 1B.

The general equation for *C* dynamics is given by


(1) \begin{equation*} \frac{dC_t}{dt} = n\phi (C) - \lambda C_{t}, \end{equation*}

where the first term presents protein generation, *ϕ*(*C*) is transcription activity, while *n* is plasmid copy number. The second term is protein degradation, and since the protein is stable, this degradation effectively happens through cell division. Before binding to DNA, C dimerizes and the dimer formation is provided by the following equations, which provide a connection between total (*C*_*t*_) and free monomer (*C*) concentrations.


(2) \begin{eqnarray*} D &=& C^2/K_{d,1}, \nonumber \\ C_t &=& C + 2D, \end{eqnarray*}

leading to


(3) \begin{equation*} C = \frac{K_{d,1}}{4}(\sqrt{1+\frac{8C_t}{K_{d,1}} } -1). \end{equation*}

Here, *D* is dimer concentration, and *K*_*d*, 1_ is the *C*-monomer dissociation constant. Mathematically, the dimerization complicates the subsequent analytical derivations since instead of rational functions (polynomials), one has to deal with square roots. This is, however, necessary for a realistic system description since *K*_*d*, 1_ is rather large (on the order of few μ*M* (33)), i.e. is on the same order as measured concentrations of R–M system components (24). Thus, a usual assumption in modeling, which posits that either monomers or dimers predominantly exist within the solution, is not justified here. Consequently, complete monomer-dimer equilibrium through Equation 3 has to be taken into account.

The main challenge is calculating the transcription activity *ϕ*(*C*) in Equation 1. There are three configurations in which *C* protein dimer can bind to promoter DNA, shown in Figure 1B. In this figure, *Z*_1_ corresponds to activation, while *Z*_2_ and *Z*_3_ correspond to repression configurations. From statistical physics, we can calculate weights for all three configurations (42), given by the following expressions:


(4) \begin{eqnarray*} Z_1 &=& C^2/(K_{d,1}K_{d,2}), \nonumber \\ Z_2 &=& (\omega C^4)/(p K_{d,1}^2 K_{d,2}^2), \nonumber \\ Z_3 &=& C^2/(p K_{d,1}K_{d,2}), \end{eqnarray*}

where *K*_*d*, 2_ and *K*_*d*, 3_ are DBS and PBS dissociation constants, respectively, *K*_*d*, 2, 3_ is tetramer dissociation constant, while

- *p* = *K*_*d*, 3_/*K*_*d*, 2_ – strength of DBS binding versus PBS,
- ω = *K*_*d*, 2_*K*_*d*, 3_/*K*_*d*, 2, 3_ – binding cooperativity between DBS and PBS,
-
$\alpha =\sqrt{K_{d,1}/K_{d,2}}$ – dimer formation parameter.

Parameters α, *p*, and ω are fixed for a given R–M system (depending on the biophysical interaction parameters) but change between different R–M systems, so we consider them *internal* system parameters. Values of these parameters, inferred from experimental data by using the expressions above, are summarized in Table 1, with details of the inference process provided in the Supplementary Material:

The transcription activity is directly proportional (with the proportionality constant *ϕ*_*m*_) to the weight of the activation configuration divided by the sum of weights of all configurations (the partition function) (42). However, there will also be a promoter leakage; that is, even in the absence of the activator C, there will be some basal promoter activity *ϕ*_*l*_. Thus, the expression for *ϕ*(*C*) becomes:


(5) \begin{equation*} \phi (C) = \phi _l + \phi _m\frac{ Z_1 }{ 1+Z_1+Z_2+Z_3}, \end{equation*}

which, after replacing expressions for statistical weights (Equation 4) becomes:


(6) \begin{equation*} \phi (C) = \phi _l + \phi _m \frac{ C^2/K_d^2 }{ 1 + (1+\frac{1}{p})C^2/K_d^2 + \frac{\omega }{p}C^4/K_d^4 }, \end{equation*}

where $K_d=\sqrt{K_{d,1} K_{d,2}}$, with the units of concentration. The Equation 1 then becomes


(7) \begin{equation*} \frac{dC_t}{dt} = n\phi _l + n\phi _m \frac{ C^2/K_d^2 }{ 1 + (1+\frac{1}{p})C^2/K_d^2 + \frac{\omega }{p} C^4/K_d^4 } - \lambda C_{t}. \end{equation*}

We do not explicitly include the equation for R dynamics since R is co-transcribed with C. Assuming no variable attenuation in the bicistronic transcript, and no variation in relative translation, their amounts should be directly proportional, i.e.


(8) \begin{equation*} R_t=K_{RC} C_t, \end{equation*}

where *K*_*RC*_ is the proportionality constant.

If we now introduce the following rescaling $\tilde{C}=C/K_d$, $\tilde{C}_t=C_t/K_d$ and divide the whole equation by *nϕ*_*m*_, we obtain the rescaled *C*-protein dynamics ($\tau =\frac{n\phi _m}{K_d}t$):


(9) \begin{equation*} \frac{d\tilde{C}_t}{d\tau } = s + \frac{ \tilde{C}^2 }{ 1 + (1+\frac{1}{p})\tilde{C}^2 + \frac{\omega }{p}\tilde{C}^4 } - \frac{\tilde{C}_t}{r}, \end{equation*}

where

-
$s=\frac{\phi _l}{\phi _m}$ – promoter leakage
-
$r=\frac{n\phi _m}{\lambda K_d}$ – overall *C* expression strength

are *external* parameters for a given R–M system, which depend both on the system and external conditions. We interpret *r* as the overall expression strength as it directly relates to the plasmid number *n* and the expression rate in *K*_*d*_ units, $\frac{\phi _m}{K_d}$, both of which contribute to increased system expression. *r* is also inversely proportional to the cell growth rate, which controls the cell proteins’ dilution (effective degradation) so that slower growth (lower λ) increases the protein expression levels. For a given R–M system, *r* can change in response to changes in the cell growth rate λ and plasmid copy number *n*. For example, different nutrient availability or stress conditions (43,44) can affect growth rate λ, while the plasmid copy number *n* can be varied in the experiment (and also in native systems) through plasmids with different replication rates (24). In the subsequent analysis, we fix *p*, ω and α for a given system based on experimental measurements (Table 1), while *r* and *s* are initially considered variable; however, where possible, we estimate *s* from experimental data, as presented in the Supplementary Material.

**Table 1.** Internal parameter values for selected R–M systems^1^

	*p*	ω	α	Reference
Esp1396I	25	130	16.9	(33)
AhdI	20	3000	$5\sqrt{2}$	(35)
EcoRV	5	1	4.2	(23)

^1^Rows correspond to the parameter values for Esp1396I, AhdI and EcoRV R–M systems (see Figure 1). The first three columns correspond to the internal parameter values, p, ω and α, which represent system-specific biophysical interaction properties related to the binding strengths and cooperativity of the C protein (see bullet points above). The last column provides a reference from which the experimental values (dissociation constants) for inferring parameter values are used.

Promoter leakage *s* is also taken as an external parameter, as it might change depending on global factors that influence transcription rates such as DNA superhelicity (45), and because in distinction to the internal parameters (*p*, ω, α), *s* cannot be inferred directly from the available biophysical measurements – though it is possible to infer it more indirectly from the available transcription activity or gene expression level measurements.

### Analytically derived stability diagram

In the Supplementary Material, we derived *r* and *s* as explicit functions of $\tilde{C}_t$ (Equations (S7) and (S8), respectively), allowing for their direct calculation as $\tilde{C}_t$ changes. This defines a stability diagram in a parametric form in *r* − *s* plane by plotting *s* versus *r* for the different parameter ($\tilde{C}_t$) values (25). The approach of deriving the stability diagram in the parametric form is of a major advantage here since the closed form dependence of *s* versus *r* (from which $\tilde{C}_t$ is eliminated) would be very hard to obtain. The derived stability diagram also fully considers the dimer-monomer equilibrium necessary for a realistic system description (see Subsection 'Model setup'). For a given R–M system, we plot a stability diagram for fixed values of the internal parameters α, *p*, and ω that characterize the system, while for a different R–M system, it is simply replotted for different values of internal parameters.

### Bifurcation diagram and cusp catastrophe surface calculation

We first generate the stability diagram using Equations (S7) and (S8) based on internal parameters for the systems shown in Table 1. We then select a value of *s* within a bistable zone from the bifurcation diagram, keep *s* constant and vary *r*. For each *r*, we numerically find $\tilde{C}_t$ as a solution to Equation S5. In monostable regions, each *r* yields a single solution for $\tilde{C}_t$. In contrast, bistable regions produce three solutions for $\tilde{C}_t$ per *r* value: two stable and one unstable. For values of *s* outside the bistable domain, following the same methodology results in a single $\tilde{C}_t$ solution for each *r*, indicating no bistability.

The cusp catastrophe surface plot is calculated similarly to a bifurcation diagram except that $\tilde{C}_{t}$ is calculated for each point in the *r* − *s* plane, so a 3D surface plot is created. Such surface folds over on itself in the region of bistability, which makes its construction numerically complicated, particularly since we work under the realistic assumption of monomer-dimer equilibrium so that stationary states correspond to zeros of non-polynomial equations. To make the cusp surface, we plot the upper surface corresponding to the stable high-steady state, the lower surface corresponding to the stable low-steady state and connect them with the in-between surface of the unstable stationary state.

### Esp1396I P.M regulation

In distinction to P.CR regulation, P.M regulation is different for all three systems. We start with Esp1396I, whose P.M regulation is schematically shown in Figure 1C. Since the binding of C dimer represses P.M, M dynamics is given by:


(10) \begin{equation*} \frac{dM_t}{dt}=n\frac{\phi _M}{\frac{D}{K_{d,M}}+1} - \lambda M_t, \end{equation*}

where *M*_*t*_ is the total methyltransferase concentration, *ϕ*_*M*_ is the maximal P.M expression rate, *D* is the concentration of C dimer and *K*_*d*, *M*_ is the dissociation constant of C dimer binding to P.M. In a steady state $\frac{dM_t}{dt}=0$, and following the rescaling in Subsection 'Model setup', we obtain:


(11) \begin{equation*} \tilde{M_t}=\frac{\phi r}{1+\tilde{C}^2 \gamma }, \end{equation*}

where *ϕ* is a multiplicative constant equal to the ratio of P.M and P.C expression rates $\frac{\phi _M}{\phi _m}$, while *r* and $\tilde{C}$ are defined as in Subsection 'Model setup'. $\gamma = \frac{K_{d,2}}{K_{d,M}}$, where from the experimentaly measured dissociation constants (33), we obtain γ = 5.1.

### AhdI P.M regulation

Regulation of P.M for AhdI is schematically shown in Figure 1A. The negative feedback loop controls the expression of P.M, where M monomer dimerizes and subsequently binds to repress P.M:


(12) \begin{eqnarray*} \frac{dM_t}{dt}&=&n\frac{\phi _M}{\frac{D_M}{K_{d,M}}+1} - \lambda M_t, \end{eqnarray*}

(13) \begin{eqnarray*} M_t &=& M + 2 D_M, \end{eqnarray*}

(14) \begin{eqnarray*} D_M &=& M^2/K_{D,M}. \end{eqnarray*}

In the expressions above, *M*_*t*_ is the total methyltransferase concentration, *ϕ*_*M*_ is the maximal P.M expression rate, *M* is the concentration of monomer and *D*_*M*_ is the concentration of M dimer. Dissociation constants for M dimer binding to DNA and dimerization are *K*_*d*, *M*_ and *K*_*D*, *M*_, respectively.

In equilibrium we have $\frac{dM_t}{dt}=0$, and by rescaling M concentrations with $\sqrt{K_{D,M}K_{d,M}}$, we obtain:


(15) \begin{equation*} \tilde{M_t}=\frac{\phi r}{1+\tilde{M}^2}, \end{equation*}

(16) \begin{equation*} \tilde{M}=\frac{\alpha _M}{4}(\sqrt{1+\frac{8\tilde{M_t}}{\alpha _M}}-1), \end{equation*}

where the multiplicative constant $\alpha _M = \sqrt{\frac{K_{D,M}}{K_{d,M}}} = 2 \sqrt{5}$, based on experimental values in (34). Constant $\phi =\frac{\phi _M\sqrt{K_{d,1}K_{d,2}}}{\phi _m\sqrt{K_{D,M}K_{d,M}}}=0.2$ is estimated from the dissociation constants in (35) and by noting that maximal P.M and P.CR promoter activities (see Equation (S1)) are approximately equal. $\tilde{M_t}$ is then determined by solving Equation 15, with $\tilde{M}$ given by Equation 16.

### EcoRV P.M regulation

EcoRV regulation is schematically shown in Figure 1D. Transcription can proceed from P.M of EcoRV only when RNA polymerase is not bound to P.CR, i.e. for the repression configurations (*Z*_2_ and *Z*_3_) of C binding in Figure 1B, or empty P.CR (statistical weight of one), so that:


(17) \begin{equation*} \frac{dM_t}{dt}=n\phi _M\frac{1+Z_2+Z_3}{1+Z_1+Z_2+Z_3} - \lambda M_t. \end{equation*}

By using the expressions for statistical weights from Equation 4 and rescaling as in 2.1, we obtain that in a steady state ($\frac{dM_t}{dt}$=0):


(18) \begin{equation*} \tilde{M_t}=\phi \, r \frac{1+\frac{1}{p}\tilde{C}^2+\frac{\omega }{p} \tilde{C}^4}{1+(1+\frac{1}{p})\tilde{C}^2+\frac{\omega }{p} \tilde{C}^4}. \end{equation*}

### Stochastic simulations of system dynamics

We perform Monte-Carlo (stochastic) simulations of Esp1396I system dynamics. The dynamics are simulated in three cases:

- *Baseline model*: We account for stochastic fluctuations in plasmid and molecule (*R* and *M*) numbers due to cell division, with constitutive gene expression – i.e. without transcriptional regulation of P.CR and P.M promoters by C. The gene expression levels (constitutive promoter activities) were chosen to yield the same equilibrium molecule levels as observed experimentally. This adjustment is essential, as fluctuations in molecule numbers depend on their absolute quantities.
- *Regulated model*: This corresponds to P.CR and P.M regulation by C, according to our model. While a rescaled version of the model was convenient in the deterministic case, stochastic simulations are sensitive to the absolute number of molecules. Therefore, in the simulations, we rescale only time (not molecule concentrations) with the cell division rate. Similar to the baseline model, specific parameters *ϕ*_*l*_, *ϕ*_*m*_ (Equation 5), *K*_*RC*_ (Equation 8) and *ϕ*_*M*_ (Equation 10) are adjusted to reproduce the experimentally measured absolute numbers of *M*, *R* molecules and M-to-R ratio (Figure 6).
- *Post-segregational model*: All plasmids are removed at the initial time, and effective molecule decay (and corresponding fluctuations) are tracked with each cell division. The initial number of molecules in this model is taken from the equilibrium simulation of the regulated model.

**Figure 3.**
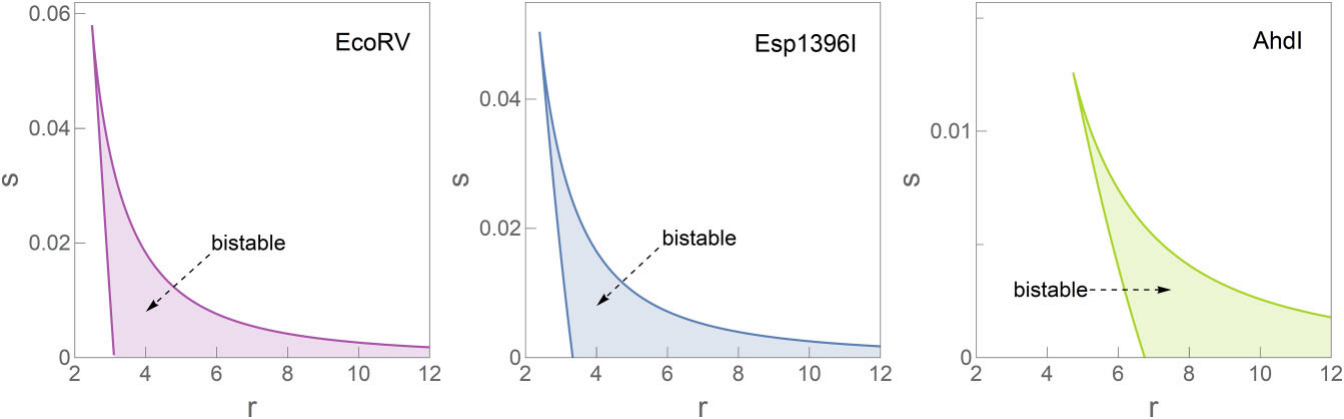
Stability diagrams for the EcoRV, Esp1396I and AhdI R–M systems. Parameters for each system are specified in Table 1. The bistable regions within each diagram are highlighted, and figures in the panel are ordered from the largest (EcoRV) to the smallest (AhdI) bistable region.

**Figure 4.**
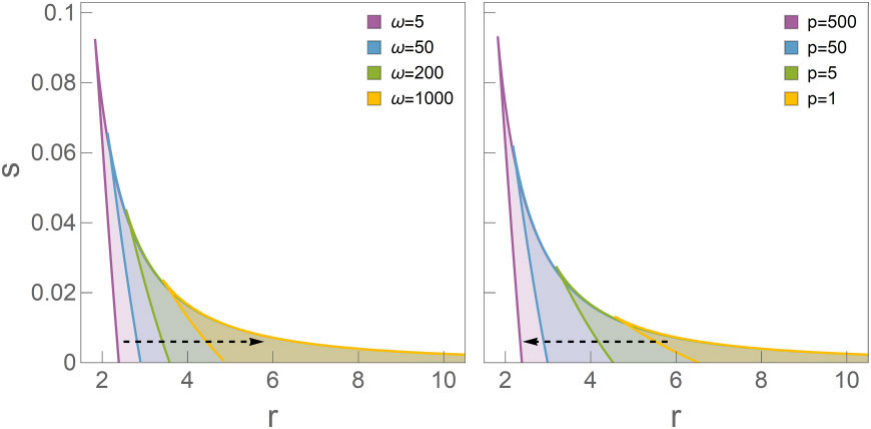
Influence of the system internal parameters on promoting bistability in Esp1396I R–M system. The bistability regions in *r* (overall C expression strength) − *s* (P.CR promoter leakage) plane are shown. The left panel illustrates the reduction of the bistability region with an increase in ω (cooperativity of C binding at the two DNA positions DBS and PBS). In contrast, the right panel demonstrates the expansion of the bistability region with an increase in *p* (the binding strength ratio between DBS and PBS). The left panel illustrates the reduction of the bistability region with an increase in ω. In contrast, the right panel demonstrates the expansion of the bistability region with an increase in *p*. The specific internal parameters are detailed in Subsection 'Model setup', Table 1.

**Figure 5.**
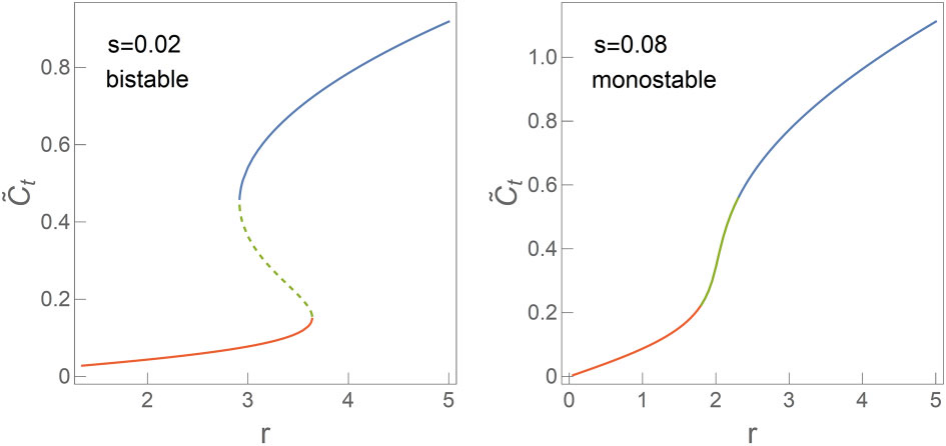
Dependence of $\tilde{C}_t$ steady state on external parameters *r* and *s* for the Esp1396I R–M system. On the left, a bistable (hysteresis) region is visible for *s* = 0.02, while on the right, a monostable region is evident for *s* = 0.08 in $\tilde{C}_t$ dynamics. Each curve segment is color-coded: blue for stable high $\tilde{C}_t$ values, red for stable low values, and green for the transitional phase between high and low stable states. Dashed and solid curves indicate unstable and bistable states, respectively. The utilized internal parameters are as in Table 1. As a reminder, $\tilde{C}_t$ is C protein dynamics, *r* is overall C expression strength and *s* is P.CR promoter leakage.

**Figure 6.**
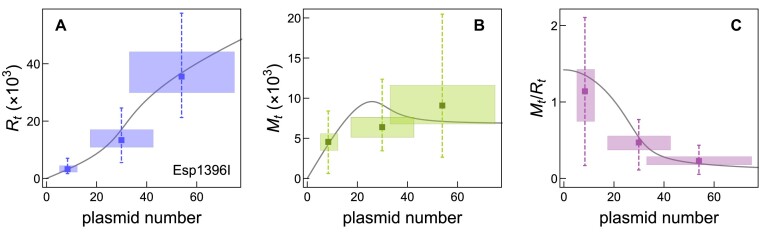
Theory versus experiment for Esp1396I R–M system. On the left, central and right panels, *R*_*t*_, *M*_*t*_ and *M*_*t*_/*R*_*t*_ dependence on the plasmid number are compared with the experimental data. Shaded rectangles indicate experimentally determined uncertainties. The rectangle width corresponds to uncertainty (±SD) in plasmid numbers. The rectangle height corresponds to the IQR, where the top and bottom edges indicate the first and third quantiles, respectively. The whiskers extend to the most extreme data points not considered outliers. Black curves are model predictions. The parameters used are stated in Subsection 'Bifurcation diagram and cusp catastrophe surface calculation', while the value of parameter *s* for which we obtained the observed agreement is *s* = 0.2.

In all three cases, we simulate 2000 cells (stochastic trajectories). Exact parameter values and reactions used in the simulations are provided in the Supplementary Tables S1–S3. The equilibrium distributions for the constitutive and regulated models are calculated after 10 cell divisions. We verified that the system is indeed at equilibrium at this time by plotting stochastic trajectories and comparing their median with the deterministic time evolution.

To assess stochastic fluctuations, the coefficient of variation (CV) was used, defined as the ratio of the standard deviation to the mean. CVs were calculated for the M-to-R ratio, a quantity directly relevant to both HGT barrier modulation and the balance between methyltransferase and restriction endonuclease in potential PSK. Since M-to-R ratio distributions notably deviate from a Gaussian distribution, we use the interquartile range (IQR) as a measure of distribution spread, and the median instead of the mean, to obtain a more robust estimate. M-to-R ratio CVs were calculated (after 10 cell divisions) and are presented for the constitutive case (no regulation), the regulated case (our model) and the experimentally measured value in equilibrium. For post-segregational dynamics, M-to-R ratio CVs were calculated after each cell division, and the median of *R* was also calculated for the same (post-segregational) simulations.

All simulations are conducted for the case of mid plasmid (approximately 30) numbers as observed in the experiment (24), corresponding to intermediate protein expression levels. At intermediate expression levels, transcriptional regulation (here, by C protein) is expected to be the dominant source of noise (46). In post-segregational dynamics simulations, trajectories where *R* dropped to zero were excluded from *M*/*R* calculations to avoid division by zero and because trajectories without *R* are not relevant for PSK. To ensure a sufficient number of non-zero *R* trajectories, we stopped the simulations after seven cell divisions.

## Results

In this section, we provide results that are essential for understanding the nonlinear dynamics of C-controlled R–M systems, specifically:

- Emergence of bistability in these systems, including which regulatory features promote and which abolish its emergence.
- Dependence of C protein and M-to-R ratio, as a crucial quantity for HGT modulation, on external factors such as bacterial growth rate, including bifurcation dependence of M-to-R ratio when the system is bistable.
- Agreement of our predictions with Esp1396I experimental data for M, R and M-to-R ratio variations with changes in plasmid copy numbers.
- Predictions of the system dynamics when all system parameters can be fixed from available experimental data (AhdI).
- Qualitative differences in system behavior for the cases where not all parameters can be mapped from available experimental data (EcoRV), allowing us to assess which properties remain robust despite parameter uncertainties.
- Effects of C protein regulation on M-to-R ratio variability across bacterial population and changes in M-to-R ratio fluctuations following R–M system removal from the cell (post-segregational dynamics).

### Stability diagrams

We start by analyzing the properties of the stability diagrams, derived analytically in Subsection 'Analytically derived stability diagram'. The analytical derivation makes the subsequent analysis straightforward, as the stability diagram for different systems (i.e., different values of the internal parameters *p*, ω, and α) is obtained by simply replotting the parametric equations of the bistability boundaries. The same applies if we want to assess how the bistability region changes when the internal parameters change. The analytical expressions provided here are a major advantage compared to the conventional numerical approach, which involves constructing a parameter space by arranging the external parameters, *r* and *s*, on a grid. That is, in the conventional approach, for each coordinate in the grid, the stability of the system is numerically evaluated; such a process then has to be arduously replicated for any change of the internal parameters (α, *p* and ω) and for all points in the *r*-*s* grid.

By employing analytical methods instead of numerical simulations, the stability boundaries can be delineated with greater efficiency, as shown in Figure 3. This figure presents the stability diagrams for *s* versus *r* for three distinct R–M systems: EcoRV, Esp1396I and AhdI. The internal parameters for all three systems, as extracted from the experimental measurements, are provided in Table 1. In all three cases, the relationship between P.CR promoter leakage (*s*) and overall C expression strength (*r*) shows that the stability boundaries vary significantly between systems. The analysis reveals that the region of bistability is the most extensive for EcoRV, while it is notably more constrained for AhdI. This already makes clear that the qualitative dynamical properties of the system are sensitive to the system’s internal parameters and can change from one R–M system to another. That is, while one system exhibits bistable behavior, the another may not, implications of which we will further assess in Discussion.

The analytical framework also facilitates a direct investigation of factors that enhance bistability. Focusing on the Esp1396I R–M system, we analyze the influence of internal parameters ω and *p* while holding others constant, which is again done by a direct plotting from Equations S7 and S8, i.e. without any numerical simulations. Figure 4 shows how varying these parameters affects the bistability domain: a decrease in ω or an increase in *p* broadens the bistable region. This observation is coherent with the principle mentioned in Introduction, that (cooperative) positive feedback promotes bistability, while negative feedback enhances steady state stability, thereby abolishing bistability. In this case, an increase in *p* favors binding to the activating (as opposed to the repression) position and consequently promotes the positive feedback, which explains the effect of *p* on the bistability region. On the other hand, increasing ω promotes the formation of the tetramer (repression) complex. Consequently, it enhances the negative feedback, which may intuitively explain the observed shrinking of the bistability region with increasing ω. Interestingly, R–M systems with the same regulatory architecture of P.CR promoter show quite different dependence on the values of these two parameters (see Table 1) – e.g. ω changes for as much as three orders of magnitude, while *p* changes less drastically but still for a factor of five. This indicates a substantial difference in the system’s quantitative properties, which may also qualitatively alter the system dynamics.

Next, in Figure 5, we construct the bifurcation diagram for the system, as described in Subsection 'Bifurcation diagram and cusp catastrophe surface calculation'. Figure 5 (left) shows bistability at a lower *s*, while Figure 5 (right) exhibits monostability for a higher value of *s*. Both panels use Esp1396I parameters, with the only difference being the value of *s*. One can understand these two figures as starting from the stability diagram (Figure 3) and making cross-sections along *r* dimension at two fixed *s* values, one corresponding to bistability and the other not.

These results indicate that bistability is favored by lower promoter leakage *s*, which can also be seen directly from the stability diagram in Figure 3. In addition to this significant influence on the system dynamics, *s* can show significant variations for different systems, as will be further analyzed below. Therefore, in addition to the changes in *p* and ω between different R–M systems, promoter leakage *s* adds another layer of variability to R–M dynamics. As discussed in Subsection 'Model setup', the overall expression strength *r* can change due to either a change in the growth rate λ (reflecting, e.g. the change in physiological conditions) or due to the change in the plasmid copy number, as will be further analyzed in the following subsection.

### Experimental comparison and predictions

For Esp1396I, experimental measurements of M amount, R amount, and M-to-R ratio changes became recently available (24). The dependence of these amounts on the plasmid copy number was measured, which in our model corresponds to changing the *r* bifurcation parameter that is directly proportional to the plasmid copy number. This provides an opportunity to compare our model predictions with the experimental data, i.e. to determine the agreement of our theoretical framework with the empirical observations (as shown in Figure 6). Free parameters in this comparison are: (i) multiplicative constants for $\tilde{R}_t$ (see Subsection 'Model setup') and $\tilde{M}_t$ (see Subsection 'Esp1396I P.M regulation') that correspond to the scales on *y*-axes on Figure 6 A and B, (ii) the common scale for the horizontal axis on all three plots in Figure 6, which corresponds to the unknown multiplicative constant for *n* in *r*, (iii) *s* that determines qualitative dynamics of the system – with the values below ∼0.05 for Esp1396I (see Figure 3), leading to bistability and, above this value, to monostable system dynamics. The fit is therefore well constrained, and as can be seen in Figure 6, we obtain a reasonable agreement with the experimental data, with the inferred *s* value of 0.2, which is well into the monostable regime.

To independently validate the inferred *s* value and further test the robustness of our model’s comparison with experimental data, we fitted the system dynamics described by Equations 3, 7 and 8 to the experimentally measured Esp1396I R dynamics from (17). This model dynamics accounts for both system regulation and the realistic C monomer-dimer equilibrium. To minimize background effects on low R molecule counts and stochastic effects, we focused on later time points with a larger number of R molecules. The resulting fit is shown in Supplementary Figure S1, from which we derived an *s* value of 0.21 – close to the value of 0.2 from Figure 6. This consistency supports the robustness of our calculations on Esp1396I dynamics.

We also note that if the Esp1396I R-M system were instead in a bistable regime, the experimentally observed nearly linear increase in *R*_*t*_ and *M*_*t*_ with plasmid number would imply that *R*_*t*_ and *M*_*t*_ lie on the red branch of the bistable region, as shown in Supplementary Figure S2A-B. This positioning suggests that the experimentally measured plasmid numbers fall below the bistable threshold. However, in this bistable configuration, the ratio *M*_*t*_/*R*_*t*_ would exhibit a slow decrease with increasing plasmid number (as shown in the red branch of Supplementary Figure S2C), which is contrary to the notable decrease in *M*_*t*_/*R*_*t*_ observed experimentally with increasing plasmid number (see Figure 6C). This discrepancy further supports the conclusion that the system resides in a monostable region.

**Figure 7.**
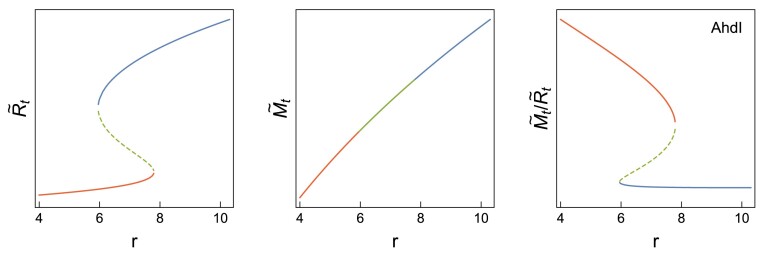
Predictions for AhdI R–M system behavior. On the left, central and right panels, $\tilde{R}_t$, $\tilde{M}_t$ and $\tilde{M}_t/\tilde{R}_t$ dependence on the plasmid number are shown, where bistable behavior is observed for $\tilde{R}_t$ and $\tilde{M}_t/\tilde{R}_t$. The vertical axis scale is provisional. The parameters used are stated in Subsection 'Bifurcation diagram and cusp catastrophe surface calculation', while *s* = 0.0043. The color code for each curve is described in Figure 5.

In Figure 7, we show the dependence of $\tilde{R}_t$, $\tilde{M}_t$ and $\tilde{M}_t/\tilde{R}_t$ on the dimensionless bifurcation parameter *r* (which is proportional to the plasmid number *n* and inversely proportional to the cell growth rate λ). For the AhdI R–M system, experimental measurements equivalent to those in Figure 6 are not yet available. However, we can infer *s* (used in Figure 7) from the experimentally measured dependence of P.CR on C protein amount reported in (15), by the procedure described in Supplementary Material. The inferred *s* value is 0.0043 for AhdI, which is well in the bistable regime, see Figure 3. This obtained result (bistable, as opposed to monostable, dynamics) is somewhat surprising given that the bistable region for AhdI is the smallest for all three systems (see Figure 3). That is, a naive conclusion, based on the stability diagram shown in Figure 3, and without the inference of *s* from the experimental data, would be that AhdI does not favor bistability. Therefore, we see that even qualitative properties of these system dynamics are inherently quantitative, i.e. determined by an interplay of several parameters. The variability obtained in the dynamic properties of these systems will be further discussed in the next section.

**Figure 8.**
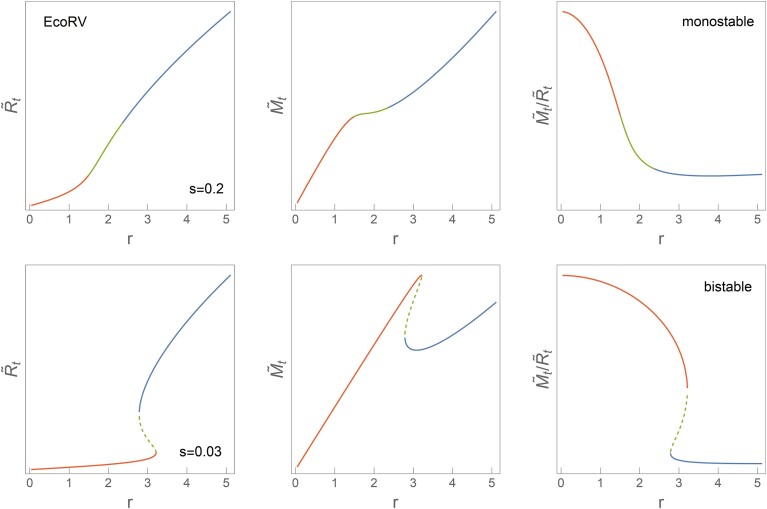
Predictions for EcoRV R–M system behavior. On the left, central and right panels, $\tilde{R}_t$, $\tilde{M}_t$ and $\tilde{M}_t/\tilde{R}_t$ dependence on the *r* are shown. In the upper (lower) panels, for *s* = 0.2 (*s* = 0.03), a monostable (bistable) behavior is predicted. The parameters used are stated in Subsection 'Bifurcation diagram and cusp catastrophe surface calculation', while the color code for each curve is described in Figure 5.

For the EcoRV R–M system, the *s* parameter value is still unknown experimentally, and we are unaware of data from which it can be estimated. Thus, depending on *s* value, the system can be either bistable or monostable, as shown in Figure 8, where small promoter leakage again leads to bistable dynamics (and large *s* values to monostability). However, for both cases and despite even possible qualitative differences in the dynamics, M-to-R ratio dependence on the overall expression strength *r* for EcoRV is predicted to have the same trend as for AhdI and Esp1396I. That is, the M-to-R ratio is lower at larger plasmid copy numbers or equivalently at slower bacterial growth rates. We will further discuss this apparently robust and arguably important system dynamical property.

### Stochastic simulations and post-segregational dynamics

In the results presented above, we analyzed the deterministic nonlinear dynamics of C-regulated R–M systems. Here, we present results from the stochastic simulations of the system dynamics, implemented through Monte Carlo simulations of a large number of cells (stochastic trajectories). Baseline stochastic effects are incorporated as fluctuations in the number of plasmids and molecules, due to random partitioning during cell divisions and molecule production. These stochastic effects are captured by the constitutive model, as detailed in the ‘Materials and methods’ section and the Supplementary Material. However, these generic stochastic effects can be significantly modulated by system regulation. For instance, negative feedback can reduce system fluctuations (47). For C-controlled R–M systems, it is challenging to assess even the qualitative direction (let alone the magnitude) of the impact of system regulation on noise, as both positive and negative nonlinear feedback loops are involved. We, therefore, also perform stochastic simulations of our full model of system regulation, which we refer to as the regulated model. By comparing the baseline (constitutive) and regulated models, we can quantitatively assess how system regulation modifies fluctuations.

**Figure 9.**
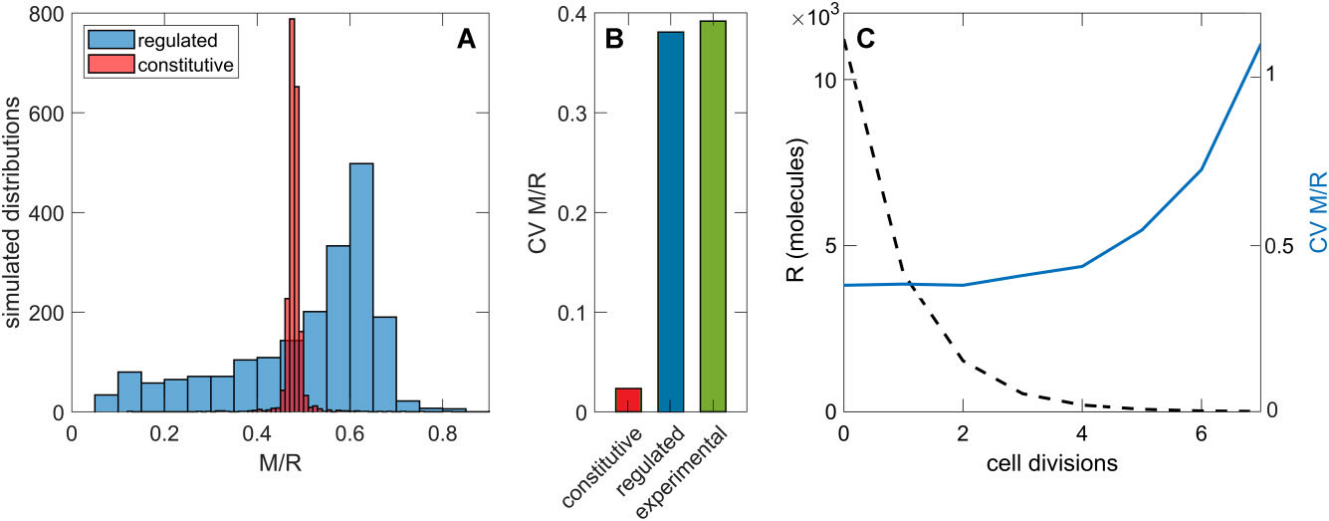
Stochastic simulations and post-segregational dynamics of the C-regulated Esp1396I R-M system. The distribution of the M-to-R ratio from stochastic simulations is shown in the left panel, comparing the regulated (blue) and constitutive (red) models. In the middle panel, the CV of the M-to-R ratio is shown for the constitutive (red) and regulated (blue) cases, along with the experimentally estimated value (green) (24). In the right panel, the dynamics of R molecule decay (black dashed curve) and the CV of the M-to-R ratio (blue curve) are shown after plasmids are removed from the cell.

We perform calculations for the Esp1396I R–M system, where experimentally estimated noise levels (24) allow us to compare simulations with empirical data. We assess the distributions from a large number of stochastic simulations for the M-to-R ratio, which is the primary quantity modulating HGT. In Figure 9A, we observe that the distribution in the regulated system is much broader (and significantly non-Gaussian in shape) than in the generic (non-regulated) case. The extent of fluctuations can be quantified by the CV (see ‘Materials and methods’).

In Figure 9B, we directly compare CVs in the constitutive (red) and regulated (blue) cases, where it is evident that regulation increases noise levels. We also observe that while fluctuations in the baseline (constitutive) case are much smaller than the experimentally estimated value (24) (green), the CV for the regulated case matches the experimental measurement. This result is both non-trivial and surprising, as one might naively expect that, when the system is present in the cell, the regulation would act to decrease noise in the M-to-R ratio to prevent fluctuations that could lead to cell death. However, we find quite the opposite: regulation substantially broadens the M-to-R ratio distribution, aligning well with experimentally measured values. We will further assess this finding in Discussion.

Furthermore, in Figure 9C, we show the results of stochastic simulations when the system (in this case, all plasmids) is removed from the cell. Qualitatively, as the number of molecules in the system decreases due to successive cell divisions, relative fluctuations (measured by CV) are expected to increase. The resulting imbalance in the M-to-R ratio could lead to the cell death, corresponding to the PSK observed in certain R–M systems (48).

However, in Figure 9C, we observe that the calculated change in CV with cell division exhibits a somewhat unusual pattern, reflecting the broad initial distribution shown in Figure 9A. The CV remains practically constant for the first four cell divisions and then starts to increase rapidly, as would intuitively be expected from the outset. By the time the CV begins to increase significantly, however, the amount of R has already decreased to low levels (shown in Figure 9C). This raises the question of whether R still retains sufficient activity to cut the host genome by the time a large enough imbalance in the M-to-R ratio is generated. In Discussion, we will relate this result to the most recent experimental findings.

## Discussion

Here, we presented a mathematical and biophysical model that encapsulates the nonlinear regulatory dynamics of bacterial R–M systems and their influence on HGT susceptibility. This theoretical framework allows a straightforward comparison of different R–M systems. This is primarily facilitated by an analytically derived stability diagram, which applies to the common regulation of P.CR by C and is characteristic of all three R–M analyzed systems. Thus, to plot a stability diagram for a different R–M system (as long as it has the same architecture of P.CR regulation), it is sufficient to change the three internal parameters of the system and replot the curves. From the stability diagram, obtaining the bifurcation diagrams is straightforward, providing dependence of the protein amounts on the crucial dimensionless parameter that encapsulates the overall expression strength and involves quantities related to the system’s physiological state.

In addition to the mathematical model allowing these predictions, it is crucial to link the model parameters with the measured experimental quantities directly. We thus provided a biophysical model of the regulation for both P.CR promoter (with the most complex regulation that may lead to bistability) and P.M promoters that are different for the three considered systems; the framework is based on statistical mechanics modeling of protein–DNA and protein–protein interactions. The standard rescaling procedure reduced complex system behavior to just five dimensionless parameters (three internal and two external), which directly relate to experimentally measured binding affinities and cooperativities. Then, we carefully assessed the literature to provide experimental parametrization of the model to the maximum extent possible. Through this, the work of many prior experiments was integrated into a coherent quantitative framework, through which comparison with experimental data and new predictions can be made. Through this procedure, we

- Showed reasonable agreement between our predictions and Esp1396I experimental data for variations of M, R and M-to-R ratio with plasmid copy number changes.
- Predicted bistable behavior for AhdI, which exhibits a substantially different P.M control from Esp1396I, through negative auto-regulation by M binding to its promoter. Similarly to Esp1396I, all relevant system parameters can be fixed from available experimental data.
- Assessed possible dynamical behavior for EcoRV, which has an entirely different control of P.M, exhibited through overlapping promoters, and where the promoter leakage (whose value determines qualitative dynamical properties) cannot be inferred from available experimental data.
- Analyzed dynamics of Esp1396I, AhdI and the two possible EcoRV behaviours, allowed us to assess which system’s dynamical properties remain robust despite parameter uncertainties and regulatory differences.

The developed model underscored the crucial role of the regulatory protein C in modulating the M-to-R ratio, which, in turn, affects the bacterial defense mechanism against invading DNA. Previous experimental studies have also suggested the important role of C in modulating the M-to-R ratio (49). Specifically, C regulation in the PvuII R–M system improved its host cell fitness since the cells with the PvuII R–M system with delated C gene were outcompeted by the wild-type cells. Smaller fitness in the absence of C regulation has been interpreted through autoimmunity, i.e. restriction of its host genome due to unbalanced R and M activities. In (24), it was found that independently changing the C amount in the system significantly influenced protection against phage infection, which is additional independent evidence supporting our finding that significant M-to-R ratio modulation can be attributed to C regulation.

Despite the differences noted above, for all three analyzed R–M systems, the M-to-R ratio showed qualitatively the same dependence on the dimensionless parameter that can be interpreted as the overall system expression strength. The expression strength is a natural bifurcation parameter, as lower expression levels are expected to promote ‘low’ C and R steady states, while higher expression levels promote ‘high’ C and R steady states. In contrast, for intermediate expression strengths, both ‘low’ and ‘high’ states may coexist (i.e., bistability may emerge). This dimensionless parameter includes the experimentally tracked plasmid copy number and, importantly, the bacterial growth rate, which depends on cell physiological conditions such as nutrient availability or stress.

The sensitivity of the M-to-R ratio to growth rates suggests a link between bacterial metabolism and defense strategy, where faster-growing cells might be more open to genetic exchange and innovation. Conversely, slower-growing cells have a lower M-to-R ratio, enhancing defense against invading bacteriophages (or channeling the incoming DNA into provision of nutrients (50,51)). Given the effects of plasmid copy number on R–M system expression, it is worth noting that growth rate also affects those copy numbers, for at least some plasmids (52). A similar effect was observed for a Type II-A CRISPR/Cas system (another BDS), whose defense components increase in stationary phase (at slow growth rate) (53). This was explained by high cell density in the slowly growing phase, which provides many opportunities for phage predation, so increasing defense against phages under these conditions might be beneficial. Moreover, Type II R–M systems and Type II-A CRISPR/Cas systems may synergize in the immune response (54), so the same obtained/observed pattern of the defense activity on growth for these systems seems plausible. R amounts in PvuII C-dependent R–M system also increase with higher plasmid copy number, consistent with the results obtained here, though M-dependence was not measured in that study (28). As an exception, in the EcoRI R–M system, it was found that the R amount decreases with higher plasmid copy numbers (55). However, M-dependence was also not measured in that study, and EcoRI has quite different regulation from the C-controlled R–M systems considered here.

It is generally hard to foresee in which way it is beneficial for bacteria to change the M-to-R ratio in terms of more efficient protection against bacteriophages – e.g. while phage absorption is lower, phage production is higher at faster growth (56,57). Overall, while the dependence of bacterial survivability on growth rate may also depend on specific ecological and environmental contexts, the robust dependence of the M-to-R ratio on growth rate predicted here deserves further experimental investigation, and the effects of HGT on growth rate are not always obvious (58). Such investigation may help us better understand how different physiological conditions modulate HGT and, consequently, how bacteria respond to different environmental and ecological challenges.

On the other hand, bistable versus monostable system behavior significantly depends on both internal (interaction) parameters and external factors (like the bacterial growth rate and plasmid copy number). This variability in R–M system dynamics may also be reflected at the genomics/evolutionary scale. While it was found that Type II R–M systems are among only a few BDSs that significantly affect HGT (underlying their significance in shaping microbial communities), this finding also changes from one bacterial species to the other (4). Our results may provide a mechanistic understanding behind this genome-level observation. While in PvuII bistability was experimentally observed (28), we predicted that it does not appear (at least not under the experimental conditions in (24)) for Esp1396I, but that it does emerge in AhdI, while in EcoRV there is insufficient experimental data to conclude on bistable versus monostable regime. Therefore, the variability predicted at the mechanistic/regulatory level, even for similar Type II R–M systems, makes the variability obtained through comparative sequence analysis plausible.

The emergence of bistability within certain parameter ranges suggests a complex regulatory mechanism that could allow both larger robustness to environmental fluctuations and diversifying response of bacterial populations to phage attacks. These possibilities are particularly intriguing when considering the ecological and evolutionary contexts in which these systems operate. As a direct consequence of bistability, fluctuations of the growth rate due to, e.g. changes in the external conditions would be buffered, allowing a more stable M-to-R ratio, as we will illustrate in more detail through the cusp surface plot below. However, bistability can also enable a subpopulation of cells to adopt a ‘defense-ready’ state with a lower M-to-R ratio. This may allow this subpopulation to survive an outburst of phage epidemics, even if the rest of the bacterial population gets wiped out. This constitutes a bet-hedging strategy, where the possibility of evading extinction due to epidemics must be balanced with the prospect of cutting the host genome (provoking autoimmunity) due to lower M-to-R ratio. This balance likely determines if it is advantageous for the R–M system to exhibit bistability, and understanding such bet-hedging tradeoffs remains an important goal for future experimental and theoretical work.

**Figure 10.**
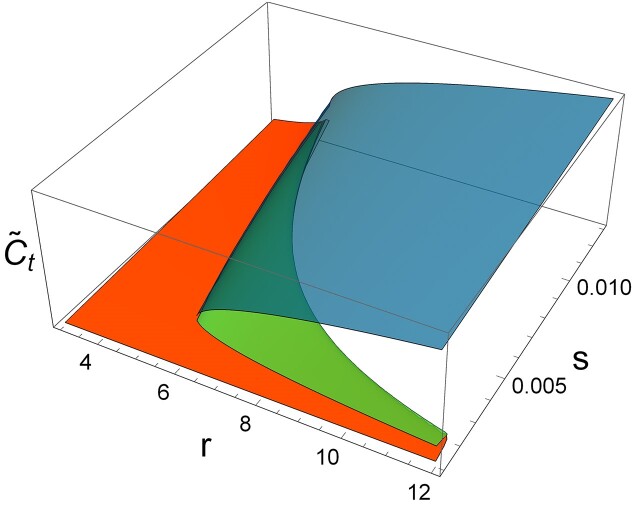
The cusp catastrophe surface plot. The surface plot is calculated for AhdI, with the parameters stated in Table 1. The three axes correspond to *r*, *s* (forming a horizontal plane) and $\tilde{C}_{t}$ on the vertical axis. The color code for each curve is described in Figure 5. The upper (blue) surface corresponds to the high R state, and the lower (red) surface corresponds to the low R state. The middle (green) surface that connects the lower (red) and the upper (blue) surface corresponds to the unstable steady state. The green surface is inaccessible due to hysteresis behavior (see the text).

The cusp catastrophe surface plot, $\tilde{C}_t$ (which is also proportional to $\tilde{R}_{t}$) is shown in Figure 10. In the bistable region, the surface – shown above *r*-*s* plane – folds over to itself. The bistable dynamics of the AhdI R–M system is shown on the plot so that the projection of the folds on *r*-*s* plane leads to the right-hand side stability diagram plot of Figure 3. Similarly, making the cross-section in the *r* direction with fixed *s* would lead to the bifurcation diagram similar to Figure 5 – note that Figure 5 was made for Esp1396I rather than AhdI (used in the cusp surface plot).

Let us assume the system is initially characterized by large R, e.g. high plasmid copy number or equivalently slowly growing cells. If the growth rate increases, the system will travel along the upper surface, reach the upper surface edge, and then drop to the lower surface. If the system were a classical engineering construction, the drop could indeed be catastrophic. There are also examples of catastrophic consequences in biology, e.g. a sudden insect outbreak could devastate the forest – see the classical model in (59), which is also presented in terms of the cusp catastrophe surface plot.

The lower surface in Figure 10 corresponds to the state where HGT is promoted, but the cell is also less protected from bacteriophage attack. However, suppose the system is just experiencing smaller fluctuations, e.g. due to environmental noise. In that case, the jump will not happen, i.e. the system will remain on the upper surface, providing robustness to smaller or even moderate perturbations in the environment. The other way around, if the system is on the lower surface (corresponding to faster growth or smaller plasmid copy numbers), it will stay there until there is a large enough perturbation that would transverse the bistable region so that the edge of the lower surface is reached – only at this point, the system will jump to the high state. Such hysteresis will then provide robustness to the external fluctuations, in addition to introducing heterogeneity in the bacterial population (discussed above).

We also used our model of system regulation to assess its stochastic dynamics. Specifically, we simulated the distribution of the M-to-R ratio across cells when the system is present and compared it to a baseline calculated in the absence of regulation but with the same generic fluctuations of molecules and plasmids. While the unregulated fluctuations are much smaller than the experimentally observed variations, predictions from our regulated model align with experimental data (24). This agreement was achieved without introducing free parameters in the stochastic simulations, as all parameters were fixed either through direct experimental measurements (internal parameters) or by fitting the deterministic model to observed mean molecule amounts (external parameters).

System regulation significantly broadens the M-to-R ratio distribution, which might seem surprising, as larger variability in the M-to-R ratio could contribute to autoimmunity – i.e. host cell killing due to an imbalance between M and R. However, increased variability in the M-to-R ratio can also diversify the bacterial cell population in terms of HGT and defense properties. This diversification may be especially relevant for Esp1396I, where we found that bistability – a distinct mechanism for diversifying bacterial populations – is likely absent. Indeed, a recent study (48) indicated that the toxicity of R.Esp1396I is lower than that of several other Type II R–M systems. Consequently, it appears that Esp1396I can sustain larger M-to-R ratio fluctuations and consequent diversity at population level without excessive risk of autoimmunity.

Another consequence of broad M-to-R ratio distributions is that, upon system removal, M-to-R ratio fluctuations remain nearly constant for the first three cell divisions. The possibility that control of R-M systems by C proteins impacts PSK was also noted before (60). In (48), both experimental and quantitative modeling results indicate that, after two cell divisions, R activity drops below the level necessary for effective cell cutting. Thus, our results are consistent with the finding in (48) that Esp1396I does not exhibit PSK. More generally, for R–M systems with higher R toxicity, the finding that M-to-R ratio fluctuations do not increase over the first few cell divisions may provide robustness against temporary perturbations that disrupt system expression without leading to unintended cell death. Conversely, if the system is indeed entirely removed, causing R and M levels to decrease irreversibly with each cell division, the M-to-R ratio will eventually increase sharply. If R activity remains sufficiently high by that time, PSK will occur. Overall, these results provide a conceptual framework for understanding an irrevocable pathway to cell death upon complete system removal while allowing for robustness in the face of transient disruptions.

As an outlook, the analysis at the cellular level points to at least two mechanisms that can generate a significant heterogeneity in the HGT barrier. This opens a venue to consider ecological and environmental contexts in understanding the evolutionary strategies of R-M system defense. Such modeling at the population level should take into account special heterogeneities since defense mechanisms like suicide are effective primarily in structured habitats due to kin selection (61,62). Furthermore, beyond serving as a model system for tight control of gene expression, the findings presented here may inspire the use of R-M systems to study the regulation of gene expression variability. To that end, experimental measurements and theoretical modeling should extend from Esp1396I to other Type II R-M systems, particularly those found to exhibit PSK.

## Conclusion

Bacterial R–M systems present a barrier to HGT, including those of pathogenic genes, by destroying unmethylated foreign DNA. Recent experimental results show that in R–M systems controlled by C, this barrier can be modulated by changing the M-to-R ratio, including changes in the restriction modification defense permeability to bacteriophage attack. We showed that control by C proteins can exhibit two qualitatively different behaviors, monostable versus bistable, depending on the value of promoter leakage, which can be inferred from experimental results. Stability diagrams derived analytically allow us to conceptualize a large body of biochemical measurements by mapping different R–M systems to their dynamical properties, which can then be directly compared. We analyzed three experimentally well-described C-controlled R–M systems, which exhibit differences in part of their regulatory architecture and substantial differences in quantitative gene regulation parameters. While these systems exhibit substantial qualitative and quantitative differences in their dynamics, M-to-R ratio dependence on the key dimensionless parameter remains largely robust, indicating that this variable is crucial for core R–M system function.

Specifically, for the investigated systems, low plasmid copy number, high growth rate, weak dimerization, and weak C protein binding to the activating position (DBS, Figure 1) lead to elevated M-to-R ratio levels and consequently promote foreign DNA acquisition. We used a cusp catastrophe surface plot to show how bistability, which appears in some C-controlled R–M systems, leads to increased robustness to environmental fluctuations. Bistability may also increase the heterogeneity of bacterial populations so that a small subpopulation of bacterial cells has a substantially different M-to-R ratio and, consequently, different potential for activity against both foreign and self DNA. In R–M systems where bistability does not emerge, we showed that system regulation may lead to large variability in the M-to-R ratio, providing an alternative mechanism for diversifying the bacterial population, with potential implications for PSK.

Overall, our study bridges (i) biophysical modeling of the system regulation, (ii) data from a number of experiments, (iii) mathematical derivation of key dynamical properties, (iv) stochastic simulations and (v) biological insight into the relation of these properties with the system function. By highlighting the intricate balance between defense mechanisms and the potential for genetic exchange, this work contributes to our broader understanding of the mechanisms by which pathogenic genes may be transmitted and the ongoing arms race between bacteria and bacteriophages.

## Supplementary Material

gkae1322_Supplemental_File

## Data Availability

Data used in the manuscript are included in the Supplement and are publicly available through the papers cited in the manuscript. The codes used to generate the results presented in the manuscript are available at https://github.com/Q-bio-Belgrade/RM_regulatory_dynamics/ and https://doi.org/10.5281/zenodo.14552173.
