# Supplementary material for: Nonlinear regulatory dynamics of bacterial restriction-modification systems modulates horizontal gene transfer susceptibility
Source: Nucleic Acids Res. 2025 Jan 16;53(2):gkae1322. doi: 10.1093/nar/gkae1322 (PMC11736437; doi:10.1093/nar/gkae1322)
Supplement: gkae1322_Supplemental_File [file gkae1322_supplemental_file.pdf]

Supplementary Material For: Nonlinear Regulatory Dynamics of Bacterial Restriction-Modification Systems Modulates Horizontal Gene Transfer Susceptibility

Magdalena Djordjevic, Lidija Zivkovic, Hong-Yu Ou, Marko Djordjevic*

S1. Parameter inference

S1.1. Inference of internal parameters from experimental data

- **Esp1396I R–M system:** Experimentally measured values are taken from [1]. The value of p is derived from the experimentally determined ratio of binding affinities to DBS and PBS, which is 200:8, directly corresponding to $p = 25$. Similarly, the parameter $\omega = 130$ is obtained from the measured 130-fold increase in binding affinity to the PBS when a C dimer is already bound to DBS. The parameter α is derived from the C dimerization dissociation constant, $K_{d,1} = 1.6\,\mu\text{M}$, and the binding dissociation constant to DBS, $K_{d,2} = 5.6\,\text{nM}$, resulting in $\alpha = \sqrt{\frac{K_{d,1}}{K_{d,2}}} = 16.9$.

- **AhdI system:** Experimentally measured values are taken from [2]. The value of p is derived from the experimentally determined ratio of binding affinities to DBS $(2 \cdot 10^7\,\text{M}^{-1})$ and the PBS $(10^6\,\text{M}^{-1})$, leading to $p = 20$. The parameter ω is obtained as the ratio of binding affinities to PBS in the presence $(3 \cdot 10^9\,\text{M}^{-1})$ and absence $(10^6\,\text{M}^{-1})$ of C dimer bound to DBS, giving $\omega = 3000$. The parameter α is derived from the C dimerization dissociation constant, $K_{d,1} = 2.5\,\mu\text{M}$, and the binding dissociation constant to the DBS, $K_{d,2} = \left(2 \cdot 10^7\,\text{M}^{-1}\right)^{-1}$, resulting in $\alpha = \sqrt{\frac{K_{d,1}}{K_{d,2}}} = 5\sqrt{2}$.

*Corresponding author. Email: dmarko@bio.bg.ac.rs

- **EcoRV system:** Experimentally measured values are taken from [3]. Here, $p = 5$ is obtained from the measured 5-fold higher binding affinity of the C dimer to DBS compared to PBS. No cooperativity in binding of C dimer between DBS and PBS was detected, resulting in $\omega = 1$. The value of α is based on the binding dissociation constant to DBS, $K_{d,2} = 115\,\text{nM}$, and the C dimerization constant, $K_{d,1} = 2\,\mu\text{M}$, giving $\alpha = 4.2$.

S1.2. AhdI promoter leakage inference

In this subsection, we infer s for AhdI from Eq. (6) in the main text by using the observed ratio between the basal (for zero C concentration) and maximal transcription activity from [4].

From Eq. (6), it is straightforward to obtain that minimal and maximal promoter activities are equal to

$$\begin{aligned} \phi_{\text{min}} &= \phi_l \\ \phi_{\text{max}} &= \phi_l + \frac{\phi_m}{1 + \frac{1}{p} + 2\sqrt{\frac{\omega}{p}}}, \end{aligned} \tag{S1}$$

leading to

$$s \equiv \frac{\phi_l}{\phi_m} = \frac{1}{\left(1 + \frac{1}{p} + 2\sqrt{\frac{\omega}{p}}\right)\left(\frac{\phi_{\text{max}}}{\phi_{\text{min}}} - 1\right)}. \tag{S2}$$

In [4], it was obtained that the ratio of maximal and minimal promoter activity for AhdI is $\frac{\phi_{\text{max}}}{\phi_{\text{min}}} = 10$. After using p and ω from Table 1 in the main text, Eq. S2 straightforwardly leads to $s = 0.0043$ for AhdI.

S1.3. Esp1396I promoter leakage inference

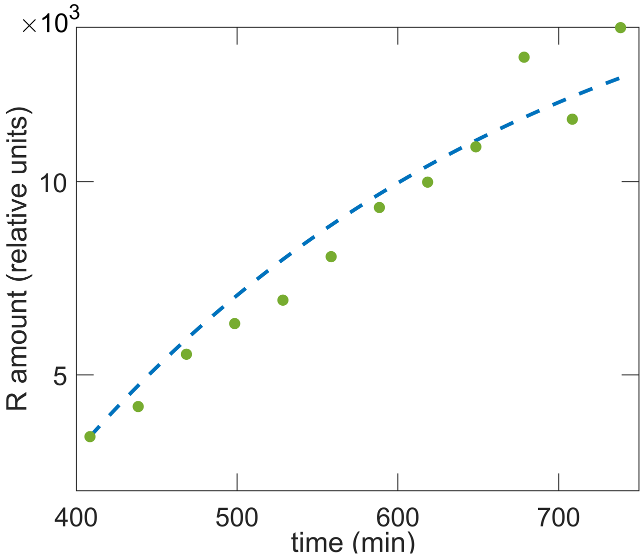

Supplementary Figure S1: **Fit of the model dynamics to the experimentally measured Esp1396I R amount over time.** Green dots represent experimental data from [5], while the blue dashed line shows the fitted model dynamics based on Eqs. 3, 7, and 8 from the main text. The model accounts for system regulation and realistic C monomer-dimer equilibrium, focusing on later time points to minimize background effects on low R molecule counts and stochastic effects. The fit was obtained by solving the system dynamics with varying external parameters (ϕ_l, ϕ_m, and K_{RC}), while all other parameters were fixed to experimentally measured values (Tables 1 and S1) and the cell division rate λ (determining R dilution/decay) was set as in [5]. The parameter space was explored using a global optimization (genetic) algorithm to identify optimal values that minimize the sum of squares error. From this fit, an s value of 0.21 was obtained.

S2. Analytical derivation of the stability diagram

We start from the rescaled form of Eq. (12) in the main text:

$$\tilde{C} = \frac{\alpha}{4}\left(\sqrt{1+\frac{8\tilde{C}_t}{\alpha}}-1\right), \tag{S3}$$

where $\tilde{C} = \frac{C}{K_d}$, $\tilde{C}_t = \frac{C_t}{K_d}$ and $\alpha = \sqrt{\frac{K_{d,1}}{K_{d,2}}}$. $\tilde{C}$ from Eq. S3 is then used in the equation for the rescaled C-protein dynamics (Eq. (9) in the main text).

To analytically determine the bistability region, two conditions have to be met:

$\tilde{C}_t$ has to be in equilibrium, i.e.,

$$\frac{d\tilde{C}_t}{d\tau} = 0, \tag{S4}$$

from which we obtain:

$$s = \frac{\tilde{C}_t}{r} - \frac{\tilde{C}^2}{1 + \left(1 + \frac{1}{p}\right)\tilde{C}^2 + \frac{\omega}{p}\tilde{C}^4}. \tag{S5}$$

In a graphical representation, the system enters a bistable region when the line $\frac{\tilde{C}_t}{r}$ becomes a tangent to the curve $\frac{\tilde{C}^2}{1+\left(1+\frac{1}{p}\right)\tilde{C}^2+\frac{\omega}{p}\tilde{C}^4}$, which mathematically corresponds to

$$\frac{1}{r} = \frac{d\left(\frac{\tilde{C}^2}{1+\left(1+\frac{1}{p}\right)\tilde{C}^2+\frac{\omega}{p}\tilde{C}^4}\right)}{d\tilde{C}_t}. \tag{S6}$$

After straightforward mathematical manipulation, this leads to

$$r = \frac{\sqrt{1+\frac{8\tilde{C}_t}{\alpha}}\left\{1+\left(1+\frac{1}{p}\right)\left[\frac{\alpha}{4}\left(\sqrt{1+\frac{8\tilde{C}_t}{\alpha}}-1\right)\right]^2+\frac{\omega}{p}\left[\frac{\alpha}{4}\left(\sqrt{1+\frac{8\tilde{C}_t}{\alpha}}-1\right)\right]^4\right\}^2}{\frac{\alpha}{2}\left(\sqrt{1+\frac{8\tilde{C}_t}{\alpha}}-1\right)\left\{1-\frac{\omega}{p}\left[\frac{\alpha}{4}\left(\sqrt{1+\frac{8\tilde{C}_t}{\alpha}}-1\right)\right]^4\right\}}, \tag{S7}$$

and by replacing Eq. S7 into Eq. S5, we finally obtain

$$\begin{aligned} s \;=\; & \frac{\frac{\alpha}{2}\left(\sqrt{1+\frac{8\tilde{C}_t}{\alpha}}-1\right)\left\{1-\left[\frac{\alpha}{4}\left(\sqrt{1+\frac{8\tilde{C}_t}{\alpha}}-1\right)\right]^4\frac{\omega}{p}\right\}\tilde{C}_t}{\sqrt{1+\frac{8\tilde{C}_t}{\alpha}}\left\{1+\left(1+\frac{1}{p}\right)\left[\frac{\alpha}{4}\left(\sqrt{1+\frac{8\tilde{C}_t}{\alpha}}-1\right)\right]^2+\frac{\omega}{p}\left[\frac{\alpha}{4}\left(\sqrt{1+\frac{8\tilde{C}_t}{\alpha}}-1\right)\right]^4\right\}^2} \\ & - \frac{\left[\frac{\alpha}{4}\left(\sqrt{1+\frac{8\tilde{C}_t}{\alpha}}-1\right)\right]^2}{1+\left(1+\frac{1}{p}\right)\left[\frac{\alpha}{4}\left(\sqrt{1+\frac{8\tilde{C}_t}{\alpha}}-1\right)\right]^2+\frac{\omega}{p}\left[\frac{\alpha}{4}\left(\sqrt{1+\frac{8\tilde{C}_t}{\alpha}}-1\right)\right]^4}. \end{aligned} \tag{S8}$$

From Eqs. S7 and S8, r and s are explicitly expressed as functions of $\tilde{C}_t$, enabling their direct calculation as $\tilde{C}_t$ varies. This yields a parametric stability diagram in the r-s plane, with s plotted against r for different values of $\tilde{C}_t$.

S3. Predicted response of the Esp1396I R–M system in a bistable regime

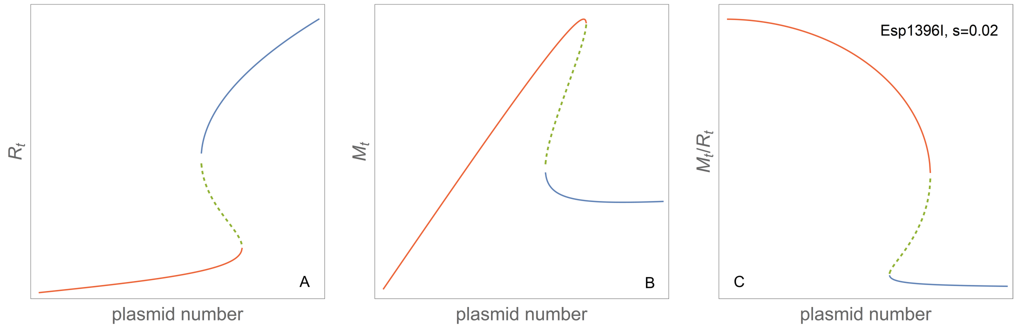

Supplementary Figure S2: **Predictions for Esp1396I R-M bistable system behavior.** The left, central, and right panels show the predicted dependence of $\tilde{R}_t$, $\tilde{M}_t$, and $\tilde{M}_t/\tilde{R}_t$ on the plasmid number for a typical value of $s = 0.02$, where bistable behavior is expected as shown in the central panel of Fig. 3 in the main text. All quantities are shown in arbitrary units and start from zero. This figure illustrates how the system would behave if it were in a bistable regime. Each curve segment is color-coded: blue for the stable high $\tilde{C}_t$ (proportional to R_t) state, red for the stable low $\tilde{C}_t$ state, and green for the transitional phase between high and low stable states. Dashed curves indicate unstable states, while solid curves represent stable states. Parameter values are provided in the Section 'Materials and methods' of the main text.

S4. Stochastic simulations

Monte Carlo simulations [6] of the system dynamics were implemented in three cases, as described in Subsection 'Stochastic simulations of system dynamics' in the main text. Below, we provide the reactions, rates, and parameters used in these simulations. In both the constitutive and regulated expression models, simulations are initiated with plasmid copy number $n = 30$, $M = 0$, and $R = 0$.

Reaction	Propensities
$n \to n+1$	n
$n \to n-1$	n
$R \to R+1$	$n \cdot \varphi_R$; $\varphi_R = 445$
$R \to R-1$	R
$M \to M+1$	$n \cdot \varphi_M$; $\varphi_M = 213$
$M \to M-1$	M

Supplementary Table S1: **Propensities for reactions in the constitutive expression model**. n represents the plasmid copy number. φ_R and φ_M denote the R and M promoter expression rates, which are constitutive, i.e., unregulated.

S4.1. Constitutive expression

A Gillespie (Monte Carlo) simulation was performed using the propensities in the table below.

Note that time is given in units of $1/\lambda$, where λ represents the cell division rate. We assume that M and R are stable within the cell, meaning the rate of protein dilution (effective decay) is equivalent to the cell division rate. Consequently, the propensities for R and M decay are $\lambda \cdot R$ and $\lambda \cdot M$, which, after rescaling by λ, reduce to R and M. Plasmids are also diluted at rate λ, so to maintain a constant mean plasmid number (here 30), the rate of plasmid replication also corresponds to λ. The simulation thus accounts for fluctuations in both plasmid and molecule (M and R) amounts due to cell divisions.

The values of φ_R and φ_M listed in the table are obtained from the experimentally measured R and M values (R_{exp} and M_{exp} [7]) as $\varphi_R = \frac{R_{exp}}{n}$ and $\varphi_M = \frac{M_{exp}}{n}$.

S4.2. Regulated expression

Internal system parameters that enter the expressions for $\phi(C_t)$ (Eqs. 6 and 3, main text) and $\phi_M(C_t)$ (Eqs. 10 and 3, main text), where $C_t = R/K_{RC}$, are derived in subsection S1.1. External parameter values, inferred from experimentally measured data [7] as described in the 'Materials and Methods' section of the main text, are provided in Table S2.

Monte Carlo simulations are performed as described in Subsection 'Stochastic simulations of system dynamics' of the main text. The reactions and their propensities follow the model for Esp1396I presented in the main text and are summarized below.

Parameter	Value
ϕ_l	400
ϕ_m	2585
K_{RC}	1043
ϕ_M	265

Supplementary Table S2: **External parameters inferred from experimental data.**

Reaction	Propensities
$n \to n+1$	n
$n \to n-1$	n
$R \to R+1$	$n \cdot \phi(C_t)$
$R \to R-1$	R
$M \to M+1$	$n \cdot \phi_M(C_t)$
$M \to M-1$	M

Supplementary Table S3: **Propensities for reactions in the regulated expression model.** All parameters are described above.

S4.3. Post-segregational dynamics

We assume that at time $t = 0$, all plasmids are removed, so that M and R molecules are stochastically diluted by cell divisions. The only reactions, therefore, are $M \to M - 1$ and $R \to R - 1$, with propensities M and R, respectively. As above, time is given in units of λ, where λ is the cell division rate. The initial number of molecules for post-segregational simulations is set by the equilibrium distributions of R and M molecules in the regulated expression model, obtained from its stochastic trajectories after 10 cell divisions (see Subsection 'Stochastic simulations of system dynamics' in the main text).

References

[1] Ball, N. J., McGeehan, J. E., Streeter, S. D., Thresh, S. J. and Kneale, G. G. (2012). The structural basis of differential DNA sequence recognition by restriction–modification controller proteins. *Nucleic Acids Res.*, **40**, 10532-10542.

[2] McGeehan, J. E., Papapanagiotou, I., Streeter, S. D. and Kneale, G. G. (2006). Cooperative binding of the C. AhdI controller protein to the

C/R promoter and its role in endonuclease gene expression. *J. Mol. Biol.*, **358**, 523-531.

[3] Semenova, E., Minakhin, L., Bogdanova, E., Nagornykh, M., Vasilov, A., Heyduk, T., Solonin, A., Zakharova, M. and Severinov, K. (2005). Transcription regulation of the EcoRV restriction–modification system. *Nucleic Acids Res.*, **33**, 6942-6951.

[4] Bogdanova, E., Djordjevic, M., Papapanagiotou, I., Heyduk, T., Kneale, G. and Severinov, K. (2008). Transcription regulation of the type II restriction-modification system AhdI. *Nucleic Acids Res.*, **36**, 1429-1442.

[5] Morozova, N., Sabantsev, A., Bogdanova, E., Fedorova, Y., Maikova, A., Vedyaykin, A., Rodic, A., Djordjevic, M., Khodorkovskii, M. and Severinov, K. (2016). Temporal dynamics of methyltransferase and restriction endonuclease accumulation in individual cells after introducing a restriction-modification system. *Nucleic Acids Res.*, **44**, 790-800.

[6] Feng, J., Kessler, D. A., Ben-Jacob, E. and Levine, H. (2014). Growth feedback as a basis for persister bistability. *PNAS*, **111**, 544-549.

[7] Kirillov, A., Morozova, N., Kozlova, S., Polinovskaya, V., Smirnov, S., Khodorkovskii, M., Zeng, L., Ispolatov, Y. and Severinov, K. (2022). Cells with stochastically increased methyltransferase to restriction endonuclease ratio provide an entry for bacteriophage into protected cell population. *Nucleic Acids Res.*, **50**, 12355-12368.
